# Relationship between Perineal Body Length and Degree of Perineal Tears in Primigravidas Undergoing Vaginal Delivery with Episiotomy

**DOI:** 10.1155/2021/2621872

**Published:** 2021-09-15

**Authors:** Suskhan Djusad, Yuditiya Purwosunu, Fadil Hidayat

**Affiliations:** Department of Obstetrics and Gynecology, Universitas Indonesia, Jakarta 10430, Indonesia

## Abstract

**Background:**

Perineal tears are one of the most common complications of vaginal delivery. Severe perineal tears can cause various morbidities. There are many factors that affect the occurrence of perineal tears. One of the major factors related to the occurrence of perineal tears is the length of the perineal body. However, until now, no research in Indonesia has concluded that the length of perineal body can predict the perineal tears.

**Objective:**

To find the relationship between perineal body length and perineal tears, so it can provide a reference on the use of perineal body length to predict severe perineal tears in vaginal delivery with episiotomy.

**Methods:**

This nested case-control study was conducted at RSUD Tangerang and RSUD Karawang in Indonesia from February to September 2017. A total of 126 primigravida patients participated in the study consecutively. The length of the perineal body was then measured and followed until the start of the second stage of labor. Afterwards, the perineal length and degree of perineal tears were assessed using unpaired *T*-test for bivariate analysis, multivariate analysis, and scoring test to predict the occurrence of third- and fourth-degree of perineal tears with power calculation (*β*) 80% and Z*β* 0.842.

**Results:**

There was a significant difference in mean length of the perineal body between the group with first- and second-degree perineal tears and the group with third- and fourth-degree perineal tears (*p* < 0.001). From the multivariate analysis, adjusted OR was 5.26 (95% CI 1.52–18.17). Score test was performed to predict the occurrence of third- and fourth-grade perineal tears. Perineal body length and head circumference could be used as predicting factors of perineal tears. Perineum length ≤ 3.0 cm and head circumference ≥ 33.5 cm posed a risk of perineal tears of third and fourth degrees (70.52%).

**Conclusion:**

The length of the perineal body has a good ability to predict the occurrence of perineal tears.

## 1. Introduction

Perineal tears are one of the complications that may occur in vaginal delivery. This complication occurs if the labor is not handled properly, and there are also accompanying risk factors during labor, such as instrumental delivery (forceps; vacuum), prolonged second stage of labor (> 60 minutes), oxytocin use, medial episiotomy, epidural use, and delivery in lithotomy or deep squatting position [1]. The defects that occur in the perineum can be assessed by examining the extent to which part of the perineum is exposed [2]. It is a major concern if the tear includes the sphincter ani complex (third- or fourth-degree perineal tears) because it will cause short and long-term morbidity.

Many studies have been performed on the perineal tears risk factors, and the results of these studies are widely known by healthcare workers, such as nullipara, prolonged second stage, vaginal delivery with instruments, medial episiotomy, persistent posterior occiput position, and macrosomia [3–5]. However, many health workers neglect patient's perineal structure. Meanwhile, the perineal body plays an important role in determining the degree of perineal tears [3, 4]. This phenomenon could happen because of the limited research related to the length of the perineal body to the incidence of perineal tears, especially in Indonesia. A previous study by Deering et al. [6] in the National Naval Medical Center, Bethesda, Maryland, USA, provided an explanation of the perineal body length associated with third- and fourth-degree perineal tears in patients undergoing medial episiotomy or operative vaginal delivery. This study is provided to confirm previous research and determine the cutoff length of perineal body which is at risk of third- and fourth-degree perineal tears, especially in Indonesia. However, we did not involve patients with medial episiotomy and operative vagina delivery.

## 2. Materials and Methods

This research was a nested case-control study on 126 primigravida patients in RSUD Tangerang and RSUD Karawang between February and September 2017. This study collected participants consecutively and was approved by The Ethical Committee of Faculty of Medicine University, Indonesia, ref.: 72/UN2.F1/ETIK/2017. Subjects were collected by consecutive sampling with inclusion criteria consisting of a term (≥ 37 weeks), live singleton pregnancy and head presentation with maximum descent of Hodge II. Exclusion criteria consisted of labor that ends with forceps/vacuum/C-section, absence of perineal tears, malpresentation, and shoulder dystocia. Primary outcome of this study is to analyze whether perineal body length can be used to predict perineal tears in primigravidas vaginal delivery. The secondary outcomes consist of prevalence of perineal tears in patients with or without mediolateral episiotomy and the relationship between birth weight, baby's head circumference, and duration of second labor phase and perineal tears. Moreover, this study aimed to sort factors contributing to increase of the risk of perineal tears. The length of the perineal body is defined as the length between the posterior of the fourchette and the midanus measured with a ruler and recorded to the nearest tenth of centimeter. In this study, the doctors measured perineal body length at rest, while the patient was in a dorsal lithotomy position during 1^st^ stage of labor. There was confirmed by a second person to minimize interobserver variability. The perineal tears are categorized as low degree (first and second grades) and severe degree (third and fourth grades). Mediolateral episiotomy was done if it is needed. Mediolateral episiotomy was done as follows: lidocaine 1% was used for local infiltration, aspiration was performed to make sure no vessel has been penetrated, infiltration was performed beneath the vaginal mucosa and skin of perineum and deep into the perineal muscle, and after waiting for two minutes, the incision site was pinched with a forceps. We performed a mediolateral episiotomy if the perineum had thinned out and the baby's head was crowning. Data was processed using IBM SPSS Statistics version 22 and three steps were conducted: descriptive analysis of the characteristics of the subject; bivariate analysis between length of the perineum body and confounding factors (birth weight, baby's head circumference, and duration of second stage) with degrees of perineal tears; multivariate analysis.

## 3. Results

From February to September 2017, we collected 126 spontaneous partus primigravida patients who had perineal tears and met the inclusion criteria. One hundred and five patients had first- and second-degree perineal tears, and 21 patients had third- and fourth-degree perineal tears. Subjects were distributed according to the action of episiotomy, and it was found that the patients who did not undergo episiotomy did not suffer third- and fourth-degree perineal tears, so further analysis was directed towards subjects who received mediolateral episiotomy treatment (Tables 1–3).

The result of bivariate analysis showed that there was a significant difference of mean perineal body length in high degree (third and fourth degrees) compared with low degree (*p* < 0.001). The length of perineal body in patients with high degree was 2.9 cm (Table 4). From the bivariate analysis, we also found that there is an association between birth weight and baby's head circumference with the incidence of third- and fourth-grade perineal tears. Lastly, a multivariate analysis of the length of perineal body, birth weight, and baby's head height was performed. Routine episiotomy is sometimes performed in belief that it reduces severe perineal tears, particularly in primiparous women. A meta-analysis by Carroli and Belizan (2004) showed that there is an increase in high-degree perineal tears associated with no episiotomy [7], while other studies showed that selective use of mediolateral episiotomy did protect against damage to anal sphincter complex. However, the fact that the no-episiotomy group did not sustain third- or fourth-degree tears at all can be because prevention of risk factors (i.e., episiotomy) does not always help predict which women will sustain perineal tears even in the presence or absence of risk factors. The study of Byrd et al showed that baby's weight is not significantly correlated with the incidence of third-degree perineal tears [8]. There is still a controversy about whether episiotomy may increase the risk for third- and fourth-degree perineal tears. Some studies showed that episiotomy increases the risks of severe perineal tears, while opposite results were also shown. This may be explained by other risk factors, such as use of epidural analgesia, birth weight, fetal presentation and position, instrumental delivery, and labor induction. [9].

Determination of cutoff value was done in the interest of performing multivariate analysis. Determination of the cutoff values of the perineum body length, birth weight, and infant's head height at risk for third- and fourth-grade perineal tears was performed using C-statistics, and the cutoff value of perineal body length was 3.05 cm with sensitivity 67%, specificity 76.2%, and AUC 83.6% (95% CI 75.8–91.4%). Cutoff value of birth weight was 3150 grams with a sensitivity of 66.7%, specificity of 58.2%, and AUC 65.1% (95% CI 51.2–78.9%). The cutoff value of the infant's head circumference is 33.5 cm with a sensitivity of 61.9%, specificity 80%, and AUC 71.2% (95% CI 57.3–85.1%). Bivariate analysis was performed to determine which variable fulfills the multivariate analysis criteria, and it was found that all variables fulfilled the criteria (Table 5).

Multivariate analysis was performed to control confounding variables (birth weight and head circumference), and it was found that having a perineal body length of ≤ 3.0 cm (adjusted OR: 5.26; 95% CI 1.52–18.18) is associated with third- and fourth-degree perineal tears if an episiotomy is performed. That is, regarding the occurrence of a rupture if an episiotomy was performed, the odds for a patient with a perineal body length of ≤ 3.0 cm is 5.26 times higher compared with a subject having a perineal body 1 mm shorter.

According to the logistic regression, the result showed the following:

Y (probability of third- and fourth-degree perineal tears = perineal tears grades III and IV) = 1/(1 + Exp (2,673–1,66*∗*perineum – 1,89*∗*head))

From the aforementioned formula, it can be concluded that, in spontaneous vaginal delivery performed by mediolateral episiotomy, the baby's birth weight is not a risk factor for third- and fourth-grade perineal tears. The length of the perineal body and size of the infant's head circumference are two almost equivalent factors in terms of the occurrence of third- or fourth-grade perineal tears, with the head circumference size being a slightly stronger risk factor (31.26% vs. 26.64%). If both of these risk factors occur simultaneously, the risk of third- and fourth-degree perineal tears is almost certain, around 70.52% (Table 6).

## 4. Discussion

### 4.1. Primary Outcomes

This study demonstrated that mean perineum length of the subjects in this study was 3.32 ± 0.4 cm. The length of the perineal body is not much different from other races previously reported according to the research by Tsai et al. [10] which included five races (Philippines, Japan, China, Hawaii, and Micronesia) and stated that race does not affect the length of the perineal body (*p*=0.42).

In this study, it was found that there is a significant difference in terms of perineal body length between subjects who had first- and second-degree perineal tears compared with third- and fourth-degree perineal tears. In the multivariate analysis, it was also found that the length of the perineal body is an independent variable that it is related to the occurrence of third- and fourth-degree perineal tears with a perineal body length of ≤ 3.0 cm with an adjusted OR of 5.25 (95% CI 1.52–18.17) for subjects experiencing third- and fourth-degree perineal tears if episiotomy was performed.

The results obtained in this study are in accordance with previous studies such as that of Rizk et al. [11], which stated that the length of the perineal body is related to the degree of perineal tears (*r* = 0.37, *p*=0.02). The difference in this study is that Rizk et al. [11] found that the perineum length is considered at risk when the perineal body length is under 40 mm, while, in this study, the risk of third- and fourth-degree perineal tears increased if the perineum body length was ≤ 3.0 cm. A research performed by Deering et al. [6] showed also the same result, but with a smaller cutoff value of 25 mm.

### 4.2. Secondary Outcomes

Aytan et al. [12] specifically evaluated the effects of episiotomy on severe perineal tears. This study concluded that, in short perineal length (<3 cm), the degree of perineal tears would be more severe when a midline episiotomy was performed. The study then recommended performing a mediolateral episiotomy if the perineal length was < 3 cm. However, it was found that, among all patients undergoing mediolateral episiotomy, the type of episiotomy did not affect the results of the study [12].

Episiotomy constituted a special part of this study as it is one of the factors related to perineal tears. WHO and Indonesia's Ministry of Health [13] stated that routinely episiotomy is not recommended anymore. However, the rate of episiotomy varies considerably. We performed mediolateral episiotomy as a standard procedure in Cipto Mangunkusumo Hospital because of the less bleeding risk and faster recovery in reported cases in the hospital. In this study, a mediolateral episiotomy was performed on 67.5% of total subjects. Rizk et al. [11] reported an episiotomy rate of 75% in preliminary primigravidas. According to the Royal College of Obstetricians and Gynecologists [14], the episiotomy rate was 8% in the Netherlands, 14% in the UK, 50% in Europe, and 99% in Eastern Europe. Jander et al. [15] revealed that the protective role of mediolateral episiotomy was not significant (*p*=0.943) although Laine et al. [16] stated that episiotomy has a protective role against the trauma of sphincter ani, especially in primipara and labor process with aids. However, episiotomy still tends to be performed on primigravidas to facilitate the course of the labor. Patients who did not undergo episiotomy did not experience third- and fourth-degree perineal tears, so further research was directed to subjects who underwent episiotomy. In this study, we analyzed several factors that could increase the risk of perineal tears in patients who underwent episiotomy.

The infant's head circumference also has an important influence in causing the incidence of third- and fourth-degree perineal tears. A study reported by Komorowski et al. [17] showed that, in episiotomy groups, the infant's head circumference was a significant independent variable (*p* < 0.001), in which an infant head's circumference of ≥33.5 cm posed a risk of third- and fourth-degree perineal tears (adjusted OR 6.5; 95% CI 2.01–21.57) [17].

Birth weight also greatly affects the incidence of perineal tears as reported by Aytan et al. [12]. It has been found that, in mediolateral episiotomy, a greater infant's birth weight with a mean of > 3755 grams will cause a higher frequency of severe tearing (*p* < 0.036). In this study, it was also found that the risk of perineal tears of degree III and IV was higher in the subjects whose infant's birth weight was ≥3150 grams (OR 2.78 (95% CI 0.87–9.15)). However, multivariate analysis in this study showed that a birth weight of ≥3150 grams is not an independent risk factor that affects third- and fourth-degree of perineal tears. Bucchave et al. [18] stated that the odds ratio of a birth weight ≥ 4000 grams was 2.6 (95% CI 1.7–3.9), and Jander et al, [15] stated that a birth weight ≥4000 grams had OR of 3.98 (95% CI 2.12–7.47), while, in this study, all subjects had a birth weight under 4000 grams which may explain why the results were not similar.

The formula for predicting third- and fourth-degree perineal tears was made based on patients who had undergone episiotomy and it was concluded that, in spontaneous vaginal delivery, if mediolateral episiotomy was performed, short perineal length of ≤ 3 cm remained a significant risk factor for third- and fourth-degree of perineal tears with a probability of 26.64%. If it was performed with an infant's head circumference ≥33.5 cm, the risk of third- and fourth-degree perineal tears was almost certain (70.52%).

Using a scoring system is important for clinicians who may face cases of third- and fourth-degree perineal tears in daily practice by measuring the length of the perineal body, so the clinicians can predict the occurrence of third- and fourth-degree perineal tears if mediolateral episiotomies are performed during labor. It would give some considerations in deciding the next action to be performed on vaginal delivery.

### 4.3. Study Limitation

The exclusion of perineal rupture may have considerably increased the incidence of severe perineal tears, that is, third- and fourth-degree tears, to 16.7% (Table 1), which is much higher than the standard literature rate of 1–3%. This may have also affected the unusually high odds ratio and prediction of perineal rupture based on head circumference and short perineum. This study is limited in the use of clinical settings due to limited samples. It would be hard to conclude with just a small number of participants that the perineal length was able to predict perineal tears.

## 5. Conclusions

The length of the perineal body has a good ability to predict the occurrence of perineal tears. Perineal body length may be a good predictor of the occurrence of perineal tears. Meanwhile, it is still limited in the use of clinical settings due to the limited number of participants. It would be hard to conclude with just a small number of participants that the perineal length was able to predict perineal tears. So, we need further research to establish the study's result. The result showed that the risk of rupture with a short perineal body length and large baby's head circumference in patients undergoing episiotomy was as high as 70.52%, so we recommended the use of mediolateral episiotomy if there is a higher risk of third- and fourth-degree perineal tears when the perineal body length is short and/or the baby's head circumference is large.

## Figures and Tables

**Table 1:** Basic characteristics.

Variable	Description (*n* = 126)
Perineal body length (cm)	3.3 ± 0.4
Birth weight (Gram)	3054.3 ± 324.9
Head circumference (cm)	32.4 ± 1.5
Duration of second labor phase (minute)	48.6 ± 13.3

*Mediolateral episiotomy*	
Yes	85 (67.5)
No	41 (32.5)

*Perineal tears*	
Grades I and II	105 (83.3)
Grades III and IV	21 (16.7)

**Table 2:** Percentage of patients who underwent mediolateral episiotomy and sustained perineal tears.

Variable	Perineal tears *n* (%)	No perineal tears *n* (%)
Mediolateral episiotomy	85 (67.5%)	0 (0%)
No episiotomy	41 (32.5%)	0 (0%)

**Table 3:** Percentage of patients who underwent mediolateral episiotomy and sustained a degree of perineal tears.

Variable	Sustaining perineal tears	
	Grades I and II (*n* = 21)	Grades III and IV (*n* = 105)
Mediolateral episiotomy		
Yes	21 (61%)	64 (100%)
No	41 (39%)	0 (0%)

**Table 4:** Relationship between risk factors and perineal tears.

Variable	Perineal tears		*p*
	Grades III and IV (*n* = 21)	Grades I and II (*n* = 64)	
Perineal body length (cm)	2.9 ± 0.2	3.3 ± 0.4	<0.001
Birth weight (grams)	3292.9 ± 335.9	3104.6 ± 291.3	0.018
Head circumference (cm)	33.6 ± 1.1	32.7 ± 1.1	0.004
Duration of second labor phase (minutes)	53.6 ± 9.7	49.8 ± 14.6	0.33

**Table 5:** Odds ratio and *p* value between risk factors and perineal tears.

Variable	Perineal tears		*p*	OR	95% CI
	Grades I and II (*n* = 64)	Grades III and IV (*n* = 21)			
*Perineal body length*					
≤3.0 cm	21	16	0.003	5.18	1.48–19.19
>3.0 cm	43	5			

*Birth weight*					
≥3150 grams	28	14	0.053	2.78	0.87–9.15
<3150 grams	36	7			

*Head circumference*					
≥33.5 cm	13	13	<0.001	6.50	1.91–22.92
<33.5 cm	51	8			

**Table 6:** Logistic regression analysis using backward method.

Variable	Value B	*p*	OR	95% CI	
				*Lower*	*Upper*
Perineal body length ≤ 3.0 cm	1.660	0.009	5.259	1.5	18.1
Head circumference ≥ 33.5 cm	1.885	0.002	6.589	2.0	21.7
Constant	–2.673				

## Data Availability

The data used to support the findings of this study are available from the corresponding author upon request.
